# Efficacy of laparoscopic subtotal gastrectomy with D2 lymphadenectomy for locally advanced gastric cancer: the protocol of the KLASS-02 multicenter randomized controlled clinical trial

**DOI:** 10.1186/s12885-015-1365-z

**Published:** 2015-05-05

**Authors:** Hoon Hur, Hyun Yong Lee, Hyuk-Joon Lee, Min Chan Kim, Woo Jin Hyung, Young Kyu Park, Wook Kim, Sang-Uk Han

**Affiliations:** 1 Department of Surgery, Ajou University Medical Center, Ajou University School of Medicine, 206 Worldcup-ro, Youngtong-gu, Suwon, 443-749 Korea; 2 Clinical Trial Center, Ajou University Medical Center, Ajou University School of Medicine, Suwon, 443-749 Korea; 3 Department of Surgery and Cancer Research Institute, Seoul National University College of Medicine, Seoul, 110-799 Korea; 4 Department of Surgery, Dong-A University College of Medicine, Busan, 602-715 Korea; 5 Department of Surgery, Yonsei University College of Medicine, Seoul, 120-749 Korea; 6 Department of Surgery, Chonnam National University Hwasun Hospital, Hwasun, 519-763 Korea; 7 Department of Surgery, The Catholic University, Yeouido St. Mary’s Hospital, Seoul, 150-713 Korea

**Keywords:** Gastric neoplasm, Laparoscopy, D2 lymphadenectomy, Advanced gastric cancer

## Abstract

**Background:**

Despite the well-described benefits of laparoscopic surgery such as lower operative blood loss and enhanced postoperative recovery in gastric cancer surgery, the application of laparoscopic surgery in patients with locally advanced gastric cancer (AGC) remains elusive owing to a lack of clinical evidence. Recently, the Korean Laparoscopic Surgical Society Group launched a new multicenter randomized clinical trial (RCT) to compare laparoscopic and open D2 lymphadenectomy for patients with locally AGC. Here, we introduce the protocol of this clinical trial.

**Methods/design:**

This trial is an investigator-initiated, randomized, controlled, parallel group, non-inferiority trial. Gastric cancer patients diagnosed with primary tumors that have invaded into the muscle propria and not into an adjacent organ (cT2–cT4a) in preoperative studies are recruited. Another criterion for recruitment is no lymph node metastasis or limited perigastric lymph node (including lymph nodes around the left gastric artery) metastasis. A total 1,050 patients in both groups are required to statistically show non-inferiority of the laparoscopic approach with respect to the primary end-point, relapse-free survival of 3 years. Secondary outcomes include postoperative morbidity and mortality, postoperative recovery, quality of life, and overall survival. Surgeons who are validated through peer-review of their surgery videos can participate in this clinical trial.

**Discussion:**

This clinical trial was designed to maintain the principles of a surgical clinical trial with internal validity for participating surgeons. Through the KLASS-02 RCT, we hope to show the efficacy of laparoscopic D2 lymphadenectomy in AGC patients compared with the open procedure.

**Trial registration:**

ClinicalTrial.gov, NCT01456598.

## Background

Since the first laparoscopic gastrectomy for gastric cancer was performed in 1994 [1], increasingly more surgeons have performed this procedure in East Asian countries such as Korea and Japan [2]. Nevertheless, the Japanese Gastric Cancer Association (JGCA) treatment guideline recommends that laparoscopic surgery for gastric cancer should not be performed as a general practice [3]. The reason for this recommendation is that despite the benefits of laparoscopic surgery, the long-term survival results from 2 multicenter randomized clinical trials (RCTs) in Japan (registered in the University Hospital Medical Information Network [UMIN] Clinical Trial Registry as UMIN000003319) and Korea (registered in the National Institutes of Health [NIH] Clinical Trail Registry as NCT0045251) have not been reported. However, the interim analysis of a multicenter RCT conducted by Korean surgeons described the safety of laparoscopic surgery for early gastric cancer (EGC) [4]. In addition, several meta-analyses have showed that laparoscopic gastrectomy with limited lymphadenectomy for patients with EGC had non-inferior oncologic outcome relative to open surgery, and a benefit in terms of faster postoperative recovery [5-7]. Based on this evidence, most experienced surgeons have applied the laparoscopic procedure in patients with EGC.

However, the use of laparoscopic surgery in patients with locally advanced gastric cancer (AGC) remains controversial. Several obstacles have been considered as the reasons for this limitation. First, extended (D2) lymphadenectomy is an essential procedure for performing curative resection in AGC patients, which requires more sophisticated surgical techniques to ensure patient safety. Owing to some limitations of laparoscopic surgery such as impossible palpation, unsecure bleeding control, among others, the experience and skill of surgeons is more important in laparoscopic surgery for AGC. Second, some researchers have expressed the concern that the laparoscopic procedure for advanced malignant disease might aggravate cancer progression via the intraoperative intraperitoneal pressure and circulating gas. However, there is no conclusive evidence to support this hypothesis to date. Taken together, the application of laparoscopic surgery in patients with locally AGC is possible if the technical and oncologic safety is ensured.

A well-designed multicenter RCT recruiting a large sample of patients is the best option to obtain clinical evidence for novel technology in the surgical field. Considering the benefits of laparoscopic surgery, such as enhanced postoperative recovery and reduced postoperative pain, the application of laparoscopic surgery will likely be extended to more patients with AGC and EGC. Currently, the Korean Laparoscopic Surgical Society (KLASS) group launched the multicenter RCT (KLASS-02 RCT; registered at www.clinicaltrials.gov as NCT01456598) to compare the oncologic and surgical outcomes between laparoscopic and open extended lymphadenectomy in patients with locally AGC. In particular, since extended lymphadenectomy in gastric cancer surgery has been regarded as a convoluted procedure, the internal validity for the surgical technique of surgeons participating in this RCT is deemed a crucial prerequisite for this surgical RCT. Therefore, the KLASS-02-QC (registered at www.clinicaltrials.gov as NCT01283893), a study conducted to standardize the procedures of laparoscopic and open extended D2 lymphadenectomy, will be performed separately [8]. Surgeons validated through the strict qualification program of the KLASS-02-QC RCT can participate. In addition, this RCT is elaborately designed to minimize the sources of bias and distortion of the results, which can be exaggerated in a surgical clinical trial. Here, we introduce the protocol of the KLASS-02 RCT comparing laparoscopic and open D2 lymphadenectomy for patients with locally AGC.

## Methods

### Objectives

The purpose of the KLASS-02 RCT is to show the efficacy of laparoscopic distal gastrectomy with extended D2 lymphadenectomy for patients who are clinically diagnosed with locally AGC, compared with conventional open subtotal gastrectomy and D2 lymphadenectomy.

### Study design

This RCT is an investigator-initiated, randomized, controlled, parallel group, and non-inferiority trial comparing laparoscopic D2 lymphadenectomy for locally AGC patients with open conventional surgery.

Before enrollment of first patient, this study was approved from the institutional review boards (AJIRB-MED-MDB-11-223) of Ajou university hospital, Soonchunhyang university hospital, Keimyung university hospital, Chonnam national university hwasun hospital, Incheon St. Mary’s hospital, Yeoeuido St. Mary’s hospital, Dong-A university hospital, Seoul national university hospital, Seoul national university bundang hospital, Yonsei university severance hospital, Yonsei university gangnam severance hospital, Ewha womans university hospital and National cancer center. All investigators progress this study in accordance with the Declaration of Helsinki [9]. An independent institutional review board of all institutions at which the participating surgeons are affiliated has approved this study. Written informed consent will be obtained from all patients before they are recruited. This RCT will be monitored by an independent data and safety monitoring committee (DSMC) organized by the Clinical Trial Center of Ajou University Hospital.

### Study population

The patient inclusion and exclusion criteria are as follows:

#### Inclusion criteria

- Patients aged >20 and <80 years
- Patients with an Eastern Cooperative Oncology Group (ECOG) performance status of 0 or 1
- Patients with American Society of Anesthesiology score of class I to III
- Patients diagnosed with gastric adenocarcinoma that is possible to be curatively resected by subtotal gastrectomy
- Patients with primary gastric carcinoma that has invaded into the muscle propria, not into an adjacent organ (cT2 to cT4a) in preoperative studies
- Patients with no lymph node metastasis or limited perigastric lymph node metastasis (including lymph nodes around the left gastric artery) in preoperative studies
- Patients who agree to participate in the clinical study through informed consent.

#### Exclusion criteria

- Patients with possible distant metastasis in preoperative studies
- Patients who underwent past gastric resection
- Patients with gastric cancer-related complications (complete obstruction or perforation)
- Patients treated by chemotherapy or radiotherapy for gastric cancer
- Patients diagnosed with other malignancy within 5 years
- Vulnerable patients
- Patients who are participating or have participated in another clinical trial within the past 6 months.

### Study protocol

The Ajou ARO (Academic Research Organization) in Ajou University Hospital will manage this clinical trial. As soon as the participating surgeons obtain informed consent from the patients, researchers entered the patient information into an web-based electrical clinical report form (eCRF;http://clintrial.ajoumc.or.kr/klass02/). The beta version of eCRF system tested at each actual site before full implementation, to ensure data capture system and to reduce user error. The eCRF system is automatically given screening number according to the sequence in which the informed consent forms are received. And if patients meet the criteria for a randomization, then eCRF system assigned at each surgery group.. Finally, the system provides an allocation number with a pre-generated randomized code for those who are selected by inclusion and exclusion criteria. A randomized block design is applied for randomization with each investigator as the stratification factor (R 2.10.1). To maintain the properties of randomization, block size is not open to the investigators. The surgeons are immediately notified of the randomization results via e-mail. After notification, the surgeons let the patients know which type of operation they will undergo. Therefore, the surgeon and patient blinding is impossible owing to the nature of surgical RCT. However, the protocol recommends that ward staff members evaluating patient outcomes be blinded, if possible. If the surgeon does not perform the operation within 30 days after recruitment, the patients are excluded from the trial. These patients will need to be re-evaluated for re-recruitment into this RCT.

#### Laparoscopic procedure

Preoperative insertion of nasogastric tube is performed depending on each surgeon’s discretion. Prophylactic antibiotics are injected within 30 minutes before skin incision. The location and number of trocars are not limited. Surgeons will examine the abdominal cavity to determine whether there is no metastatic lesion or if the gastric cancer is resectable. If the patient has unexpected metastatic lesions or the surgeon decides that curative resection of tumor is impossible, the operation is stopped and the patient is excluded from this RCT. Washing cytology could be performed by inserting 50 cc of saline solution into the pelvic cavity. Then, the surgeon begins the D2 lymphadenectomy including total omentectomy. The guideline for D2 lymphadenectomy in locally AGC patients is shown in Table 1. If surgeons make an additional abdominal incision to control bleeding or for any other reason before finishing the laparoscopic D2 lymphadenectomy, they will record this situation as “conversion to open” in the eCRF system. After lymphadenectomy, the reconstruction methods are not limited in this RCT. The surgeon can perform one of the Billroth-I, Billroth-II, or Roux-en Y methods for reconstruction, which can be carried out by minilaparotomy. Hand-sewing or using staplers for anastomosis are not limited, and drain insertion is left to the discretion of each surgeon.

**Table 1:** **Guidelines forof D2 lymph node dissection for locally advanced gastric cancer**

1. Total omentectomy	4d
2. Division of the left gastroepiploic artery	4sb
3. Appropriate extent of No. 6 lymph node (LN) dissection	6
4. Appropriate extent of No. 5 LN dissection	5
5. Appropriate extent of No. 12a LN dissection	12a
6. Appropriate extent of No. 8a LN dissection	8a
7.Appropriate extent of No. 9 LN dissection (resection of the celiac plexus is not necessary)	9
8. Appropriate extent of No. 7 LN dissection	7
9. Appropriate extent of No. 11p LN dissection	11p
10. Prevention of pancreatic injury during suprapancreatic LN dissection	
11. Appropriate extent of No. 1 and 3 LN dissection	1, 3

LN, lymph node.

#### Open conventional procedure

The open surgery procedure is similar to that of laparoscopic surgery, with the exception of lymphadenectomy performed under direct view.

#### Postoperative care

The ward staff will evaluate the patients every morning and afternoon for the presence of any issues affecting the patients’ recovery. The degree of pain, diet schedule, and gas out were daily recorded until discharged, and laboratory findings are recorded on the first and fifth postoperative day. After surgery, the surgeon can progress the diet schedule from sips of water to a soft diet according to the patient’s condition. If the patients are in good condition 2 or 3 days after starting a soft diet and do not have complaints regarding their status, they can be discharged from the hospital.

After fully recovering from surgery, patients diagnosed with Stage II (except T1) or Stage III cancer in the final pathology report will be recommended for adjuvant chemotherapy based on 5-fluorouracil administration. Adjuvant chemotherapy will be started from 4–6 weeks postoperatively, if the patients’ general condition is suitable for chemotherapy.

After curative resection, all patients will be regularly evaluated for disease recurrence postoperatively every 3 or 6 months over a 3-year period. Abdominopelvic computed tomography (CT), serum tumor markers (CEA and CA19-9), and other parameters can be included in studies for regular follow-up.

### Participating surgeons and quality control

To participate in this RCT, surgeons will be validated through a separate clinical study (registered at www.clinicaltrials.gov as NCT01283893). Surgeons submit unedited videos of their laparoscopic and open conventional D2 lymphadenectomy procedures, which are reviewed by international experts. Finally, the review committee decides whether the surgeons are validated and can join the RCT.

After starting recruitment in this RCT, surgeons will record an unedited video of the laparoscopic operation and a photo of the open surgery for every 10 recruitments. The steering committee can request submission of these data for evaluation of safety of surgeons showing frequent severe side effects.

### Outcome measurements

The primary endpoint of the KLASS-02 RCT is non-inferiority in the 3-year relapse-free survival rate after laparoscopic subtotal gastrectomy and extended lymphadenectomy for locally AGC compared with open conventional surgery. To measure this endpoint, the criteria for recurrence are suggested in detail. Recurrence can be detected on regular follow-up studies such as abdominopelvic CT in patients without specific symptoms through a formal radiology report. If the results of follow-up studies are suspicious, whole-body positron emission tomography-CT, magnetic resonance imaging of the liver, or laparoscopic exploration can be performed to confirm recurrence. Otherwise, patients can attend follow-ups at shorter intervals than the planned schedule. Patients with specific symptoms, such as abdominal mass, weight loss, or intestinal obstruction, which might coincide with recurrence, should be evaluated for recurrence immediately regardless of the follow-up schedule. Surgeon should report recurrence in enrolled patients by entering this information into the eCRF as soon as the recurrence is confirmed.

Secondary outcomes include postoperative morbidity and mortality, postoperative recovery, and quality of life. Complications are divided into early and late morbidities depending on time of occurrence. Early morbidity is defined as surgery-related complications occurring within 21 days postoperatively, and includes the events listed in Table 2. Complication severity is classified according to grading system suggested by Dindo D et al. [10]. Regarding postoperative morbidity, the operative time and blood loss volume are also recorded. In cases of conversion from laparoscopic to open surgery, the reason for conversion should be explained. The events related to late morbidity, which occur after the 28th postoperative day, are listed in Table 2.

**Table 2:** **Classification of morbidity in the present study**

Early morbidity	Late morbidity
0: No complications	1: Intestinal obstruction
1: Wound infection	2: Stenosis
2: Fluid collection or abscess	3: Chronic wound complications
3: Intra-abdominal bleeding	4: Others
4: Intraluminal bleeding	
5: Postoperative ileus	
6: Anastomosis stenosis	
7: Leakage	
8: Pancreatitis or fistula	
9: Pulmonary	
10: Urinary	
11: Renal	
12: Hepatic	
13: Cardiac	
14: Endocrine	
15: Others	

To evaluate overall survival as one of the oncologic outcomes and surgery-related mortality, all deaths of recruited patients during the RCT will be reported immediately, and the cause of death will be recorded.

For postoperative outcomes designated as secondary endpoints, the postoperative diet schedule, recovery of bowel movements, and pain scale will be investigated every morning before hospital discharge. Serum levels of whole blood leukocytes, hemoglobin, amylase, creatinine, and albumin are recorded as biochemical outcomes twice during recovery. Finally, the length of hospital stay and readmission will be evaluated.

To investigate the quality of life, the questionnaire suggested by the European Organization for Research and Treatment of Cancer (EORTC) will be used with web-based permission from EORTC. Patients are requested to complete the EROTC-QLQ 30 and STO 22 questionnaires perioperatively and at 4 weeks and 1 years postoperatively.

### Sample size calculation

The primary endpoint of this RCT is 3-year relapse-free survival of patients diagnosed with locally AGC. In an RCT conducted by Sakuramoto et al. [9], the 3-year relapse-free survival of gastric cancer patients who received TS-1 adjuvant chemotherapy and were diagnosed with stage II or III cancer on pathology from curative gastrectomy with D2 lymph node resection was 72% (hazard rate = 0.11). Therefore, the hazard rate of patients who underwent open conventional surgery in the control condition was 0.11 in the 3 year. In addition, the margin of non-inferiority assumed a hazard ratio (HR; hazard rate of group A/hazard rate of group B) of 1.43 according to the study design in the Sakuramoto et al., in which the HR of the surgery-only group was compared with that of the adjuvant group. The null hypothesis was assigned as HR ≥ HR_0_, and the alternative hypothesis as HR < HR_0_. Type I error was set at 0.25 (one-sided) with 90% power, and the sample size was calculated using the log-rank test for non-inferiority (PASS 12. NCSS, LLC. Kaysville, Utah, USA. www.ncss.com.).

As a result, a total of 1,050 patients (525 patients per group) with 330 target event as recurrence are required when we consider a 10% dropout rate.

### Safety assessment for early morbidity

When the safety analysis group reaches 484 patients, analysis will be performed to evaluate the safety of laparoscopic subtotal gastrectomy with D2 lymphadenectomy. Considering the reports of Sano et al. [11] and Deguili et al. [12], the complication rate of standard gastrectomy with D2 lymph node dissection was estimated at 20.9% and the margin of non-inferiority for the complication rate was assumed to be 12%. With a type I error of 0.025 (one-sided) and 90% power, 242 patients in each safety analysis group are required. When a total of 484 patients are enrolled in the safety analysis groups, the steering committee will decide whether this trial would be continued according to the results of this safety analysis.

### Interim analysis

When the number of target event as recurrent cases reaches half the expected number of the calculated sample size, we will perform an interim analysis to identify if clear evidence exists that laparoscopic surgery is inferior to open surgery, and should not be used. The interim analysis will be conducted by the Haybittle-Peto interim monitoring boundary method, with type I error set at 0.001 according to the previous report of Freidin et al. [13].

### Patient groups for statistical analysis

**Figure 1:**
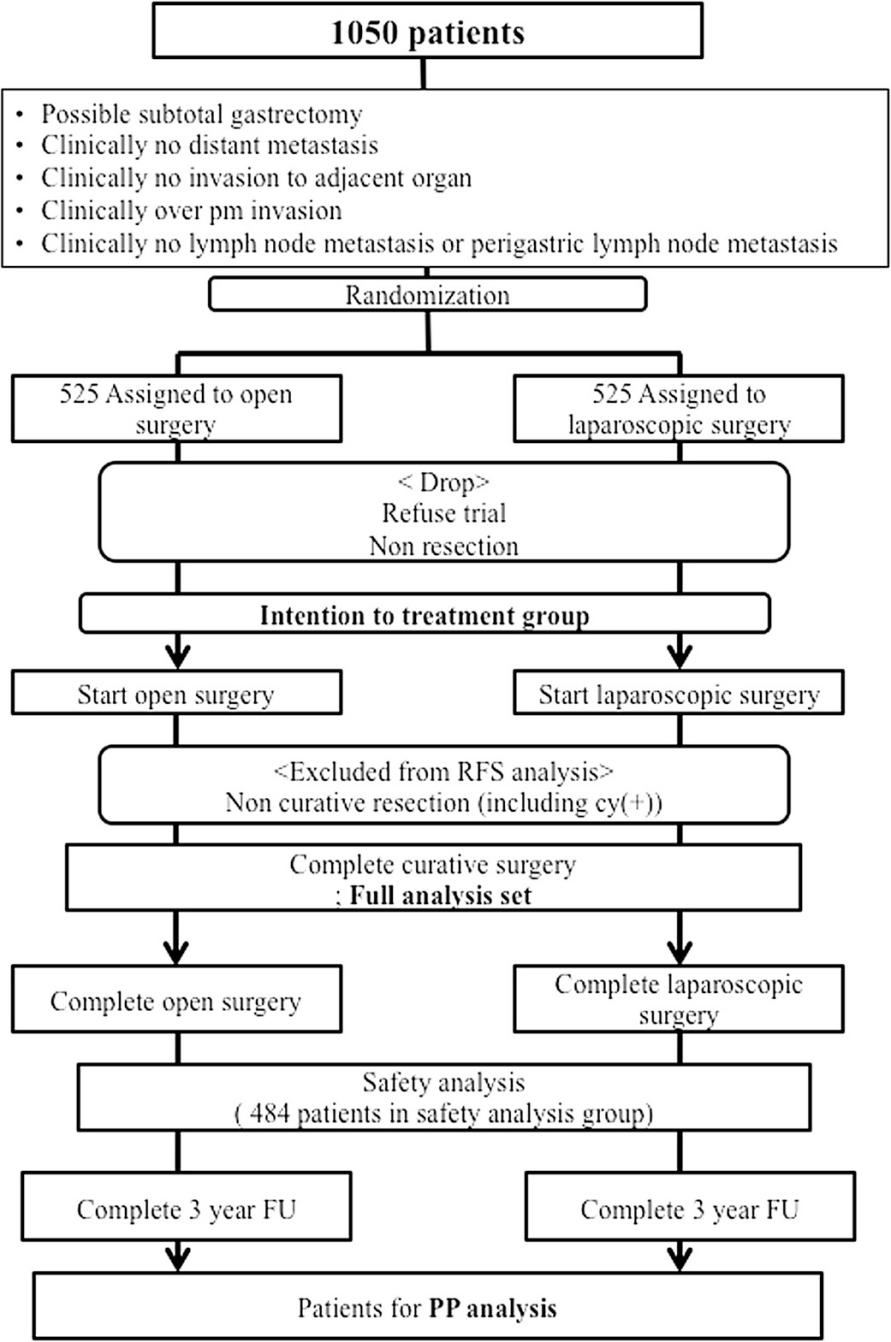
Overview of the Korean Laparoscopic Surgical Society (KLASS)-02 randomized controlled trial (RCT) design. RFS; relapse free survival, FU; follow up, PP; per protocol.

The efficacy for the primary and secondary outcomes will be evaluated in different groups of patients according to the surgical results. Except for the patients who are not undergoing surgery or have unresectable tumors, all patients will be included in the intention-to-treat (IIT) group. The postoperative recovery, morbidity and mortality, quality of life, and overall survival will be evaluated for the patients in IIT group. The 3-year relapse-free survival will be analyzed for patients in the full analysis set (FAS) group. ITT group patients who cannot undergo curative resection and have synchronous tumors or distant metastases un-defined at the screening step, will be additionally excluded from the FAS group. If patients did not comply with protocol because of various reasons like as conversion of surgical type, total resection and follow up loss, then they will be excluded from the per-protocol (PP) group. When the difference between the FAS and PP groups exceeds 10% of the total recruitment number, statistical analysis with the PP group patients will be performed. This RCT is schematically described in Figure 1. For safety analysis, patients in FAS group are examined with their actual treated surgery.

For analysis of relapse-free or overall survival, Kaplan-Meier curve analysis with log-rank tests will be used. To investigate the different proportions of patients with morbidity, mortality, or other categorical data between 2 groups, chi-square or Fisher’s extract tests will be applied. Continuous variables such as length of hospital will be evaluated using student t- or Mann–Whitney U tests. The level of significance will be set at 5%.

## Discussion

To the best of our knowledge, this study is the first multicenter randomized clinical trial recruiting a large number of patients to compare laparoscopic D2 lymphadenectomy with open conventional lymphadenectomy in patients preoperatively diagnosed with locally AGC. Through this clinical trial, we aim to show the non-inferiority in the 3-year relapse-free survival rate of patients undergoing laparoscopic procedures compared with open surgery as the primary end point. In addition, laparoscopic surgery is expected to show comparable surgical outcomes such as morbidity and mortality, and improved postoperative recovery. Particularly, we sought to design the trial to overcome the challenges confronting surgical clinical trials while following the general principles of multicenter RCTs.

In general, multicenter RCTs should be designed according to specific principles such as evidence-based calculation of sample size, concealment of randomization, interim analysis, ITT analysis, and blinding to the type of intervention to avoid bias. Our protocol was established by following these general principles as closely as possible. However, blinding of the surgeons and patients to the intervention is impossible in most surgical clinical trials. In particular, when comparing laparoscopic surgery with conventional laparotomy as in the present RCT, blinding between 2 surgical procedures cannot be achieved due to the technical difficulty. In some previous surgical RCTs, postoperative dressings were applied using the same methods for patients who underwent both laparoscopic and open cholecystectomy, thus the type of intervention was blinded to patients and ward staff [14,15]. However, it is unlikely that perfect blinding using the same dressing is achieved, and blinding patients to the type of surgery might cause ethical issues. Non-blinding of staff investigating the outcomes will likely cause subjective measurement of them. To minimize the bias due to non-blinded randomization in the present RCT, we suggested the objective outcomes as endpoints. Therefore, the criteria for recurrence, the primary endpoint of this RCT, were described in detail.

Our clinical trial was proposed at an opportune moment in the development of gastric cancer surgical approaches. To date, laparoscopic surgery has been described as a revolutionary procedure to minimize the trauma in various fields of surgery, including gastric cancer surgery. Laparoscopic limited lymphadenectomy (D1 or D1+) has been widely performed by experienced surgeons as a treatment for EGC, but not AGC [5,6]. The canonical procedure for locally AGC is gastric resection with extended lymphadenectomy (D2), as the long-term results of several RCTs have reported superiority in the survival rate of patients who underwent D2 lymphadenectomy relative to that of limited lymphadenectomy (D1) [16,17]. In contrast to limited lymphadenectomy, D2 lymphadenectomy requires dissection of the groups of lymph nodes around major vessels such as the hepatic (LN #8) and splenic (LN #11p) arteries and the portal vein (LN#12a). Owing to limitations of the laparoscopic view for these lesions, the principle of surgery for patients with locally AGC is D2 lymphadenectomy with open laparotomy. However, a recent case-matched study reported by the KLASS group showed that there was no difference in long-term survival rates between laparoscopic and open conventional gastrectomy in AGC patients [18]. Moreover, several non-randomized clinical studies have shown the oncologic feasibility and technical safety of laparoscopic D2 lymphadenectomy for AGC [19-21]. Particularly, experienced surgeons have claimed that the advanced laparoscopic instruments and imaging system enables them to dissect the groups of lymph nodes in extensive regions such LN#8, #11p, and #12a. Considering the conclusive benefit of laparoscopic surgery in postoperative recovery, expansion of laparoscopic surgery is inevitable as long as the clinical evidence is clarified. Therefore, this multicenter RCT investigating the efficacy of laparoscopic surgery in patients with locally AGC is warranted to confirm the utility of laparoscopic surgery in patients with gastric cancer.

Although a clinical trial of laparoscopic D2 lymphadenectomy seems feasible based on previous reports, we could not ignore the internal validity of the laparoscopic skill of the surgeons participating in this RCT. This issue could be problematic, as the procedures will be performed by numerous surgeons. Therefore, we established several rules to avoid problems related to internal validation. First, only surgeons who had performed ≥50 laparoscopic gastrectomies for gastric cancers and were affiliated with experienced institutions could participate in this RCT. This principle is based on previous reports in which laparoscopic surgery for gastric cancer required some experience to overcome the learning curve [22-24]. Second, we performed a separate clinical study to validate the surgeons who wish to participate in this RCT (registered at www.clinicaltrials.gov as NCT01283893) [8]. Experts review the videos submitted by candidate surgeons, and the committee decides whether they can participate in this study based on the reviewers’ results. Third, the participants will submit videos of their laparoscopic surgeries and photos of conventional open surgery every 10 recruitments for the committee’s review. These efforts to maintain internal validity will be helpful to ensure that meaningful results are obtained from this RCT.

The safety of laparoscopic D2 lymphadenectomy should be emphasized, because this study is the first multicenter RCT to evaluate its efficacy. In our RCT, morbidities related to surgeries will be investigated separately during early and late postoperative periods after surgery. Since most late complications such as reflux and intestinal obstruction are mainly related to reconstruction methods regardless of the type of laparoscopic or open laparotomy, we eventually focused on early postoperative morbidity to evaluate safety. When the number of patients recruited in this RCT reaches 484, the data related to early postoperative morbidities will be analyzed to determine whether laparoscopic surgery for D2 lymphadenectomy is less safe compared with open surgery. The number of patients required for this safety analysis was calculated according to previous RCTs, which reported that the complication rate of D2 lymphadenectomy was 20.9% in an RCT performed by Japanese surgeons, and the limitation of non-inferiority for complications of D2 lymphadenectomy compared with D1 was 12.0% in another Italian RCT [11,25]. This protocol includes a plan to stop recruitment and discontinue this RCT if the results of the safety analysis show significant inferiority in the safety of laparoscopic groups relative to the conventional group.

In conclusion, the KLASS-02 RCT was designed to show the efficacy of laparoscopic D2 lymphadenectomy in AGC patients compared with the open procedure. We attempted to highlight the principle of surgical clinical trials, and the internal validity of surgeons participating in this RCT was considered. Finally, we hope to suggest our RCT to other researchers who wish to conduct a well-designed, organized surgical clinical trial.

## Abbreviations

JGCA: Japanese Gastric Cancer Association
RCT: Randomized clinical trial
EGC: Early gastric cancer
AGC: Advanced gastric cancer
KLASS: Korean laparoscopic gastrointestinal surgery study group
ARO: Academic Research Organization
DSMC: Data and safety monitoring committee
ARO: Academic Research Organization
CT: Computed tomography
EORTC: European Organization for Research and Treatment of Cancer
IIT: Intension to treatment
FAS: Full analysis set
PP: Per protocol
